# Qingchang Huashi granule ameliorates experimental colitis via restoring the dendritic cell-mediated Th17/Treg balance

**DOI:** 10.1186/s12906-020-03088-y

**Published:** 2020-09-23

**Authors:** Jia Jia, Kai Zheng, Hong Shen, Jiangyi Yu, Ping Zhu, Shihai Yan, Yi Xu, Lei Zhu, Yuelin Lu, Peiqing Gu, Wan Feng

**Affiliations:** 1 Department of Endocrinology, Affiliated Hospital of Nanjing University of Chinese Medicine, No. 155 Hanzhong Road, Nanjing, 210029 China; 2 Department of Gastroenterology, Affiliated Hospital of Nanjing University of Chinese Medicine, No. 155 Hanzhong Road, Nanjing, 210029 China; 3 Department of Colon and Rectal Surgery, Affiliated Hospital of Nanjing University of Chinese Medicine, No. 155 Hanzhong Road, Nanjing, 210029 China; 4 Laboratory of Pharmacology, Affiliated Hospital of Nanjing University of Chinese Medicine, No. 155 Hanzhong Road, Nanjing, 210029 China

**Keywords:** Ulcerative colitis, Chinese medicine, T helper 17, Regulatory T cells, Dendritic cells, Immunological tolerance

## Abstract

**Background:**

The balance between T helper 17 (Th17) cells and regulatory T cells (Tregs) is involved in immunological tolerance. Destruction of immunological tolerance by dendritic cell (DC)-mediated T cells is involved in the pathogenesis of ulcerative colitis (UC). Qingchang Huashi granule (QCHS) has been confirmed in the treatment of UC involved by inhibiting the activation of DCs. The aim of this study was to investigate the mechanism through which QCHS restores the Th17/Treg balance by modulating DCs in the treatment of UC.

**Methods:**

The effects of QCHS on Th17 cells, Tregs and DCs were detected in a 2,4,6-trinitrobenzene sulfonic acid (TNBS)-induced experimental colitis model. Furthermore, we injected QCHS-treated DCs into colitis model to test whether QCHS modulates the Th17/Treg balance via DCs. Tregs and Th17 cells were analyzed by FACS. IL-10, IL-17, and Foxp3 were measured by ELISA, Western blot and qRT-PCR.

**Results:**

Both QCHS and QCHS-treated DCs improved colonic histopathology, diminished Th17 cell differentiation and inhibited IL-17 production while promoting CD4^+^CD25^+^Foxp3^+^ Treg differentiation and augmenting IL-10 and Foxp3 expression in colitis mice. Additionally, QCHS reduced CD86 and MHC-II expression on DCs, decreased IL-12 production ex vivo and restored the Th17/Treg ratio in the colitis model.

**Conclusion:**

The findings of this study indicate that QCHS ameliorates TNBS-induced colitis by restoring the DC-mediated Th17/Treg balance.

## Background

Ulcerative colitis (UC) is an idiopathic inflammatory bowel disease (IBD) characterized by chronic relapse and a remitting clinical course [1]. IBD has become a global disease in the past few decades. The prevalence of UC is increasing in developed countries, although the incidence is stable [2]. The prevalence and incidence of UC are lower in the Asia-Pacific region than in the West, but the incidence of UC in newly industrialized countries has increased steeply, especially in many parts of the Asia-Pacific region [3]. Important symptoms of UC include bloody diarrhea, mucous, urgency, tenesmus, abdominal cramping and weight loss. All of these symptoms impair health-related quality of life in patients and bring a substantial burden to these individuals [4]. IBD has been classified as a prototypically complex disease [5], in which biological complexity arises from intricate interactions among multiple factors, such as the environment, genes, microbiota and diet, and the precise etiology of UC remains unknown [6].

The exact pathogenesis of IBD is still unknown [7]. UC is a disease characterized by various genetic abnormalities that lead to overly aggressive T cell responses, which result in cytokine production, increasing the lymphocyte homing response, and recruitment of high numbers of T cells to the intestinal mucosa [8]. T helper (Th) 17 cells are a type of effector T cell subset, an important proinflammatory cell subset that is differentiated from naive T cells and strongly associated with inflammatory responses [9]. Th17 cells promote inflammation progression with specific secretion of interleukin (IL)-17, which activates and recruits neutrophils and macrophages to intestinal mucosa and induces inflammatory responses and tissue damage [10]. Regulatory T cells (Tregs) are a special type of T cells that have a critical role in maintaining peripheral immunological tolerance [11]. Tregs produce the cytokine IL-10, which regulates the production of inflammatory cytokines. Enhancing Treg function can protect against experimental colitis [12]. The transcription factor Forkhead box P3 (Foxp3) is critically involved in the development and function of Tregs [13]. The percentage of CD4^+^CD25^+^CD127^low^Foxp3^+^ Tregs decreased in patients with UC [14], while Th17 cells are increased in the lamina propria of patients with UC [15]. Th cells and Treg-related cytokines are implicated in UC [16]. The interaction between Th cells and Tregs is critical to the induction and regulation of immunological tolerance. The loss of homeostasis between Tregs and Th17 cells leads to aberrant immune responses in UC [17].

Dendritic cells (DCs) are a type of antigen-presenting cells that have an important role in the adaptive immune system. The initiation and control of lymphocyte responses depend on the interaction of T cells with DCs [18]. Most of DCs in the body in an immature status. After activation, mature DCs move to the lymphatic tissue to polarize naive T cells to differentiate into Th cells and reduce the differentiation of Tregs [19].

The use of complementary and alternative medicine is common in patients with IBD. The most commonly used complementary and alternative medicine treatment in most surveys is traditional Chinese medicine (TCM) [20]. Chinese herbal medicine is useful in alleviating symptoms in patients with UC, such as diarrhea and abdominal pain [21]. Qingchang Huashi granule (QCHS) is a Chinese herbal granule for active UC treatment. QCHS was approved by the Jiangsu Food and Drug Administration and manufactured by the Affiliated Hospital of Nanjing University of Chinese Medicine. The granule includes *Rhizoma Coptidis* (Huanglian), *Radix Aucklandiae* (Muxiang), *Radix Scutellariae* (Huangqin), *Radix Sanguisorbae* (Diyu), *Radix Pulsatillae* (Baitouweng), *Radix Angelicae Sinensis* (Danggui), *Radix Ampelopsis* (Bailian), *Radix Paeoniae Alba* (Baishao), *Cinnamomum cassia* (Rougui), and *Radix Glycyrrhizae* (Gancao). The efficacy of QCHS has been confirmed in subsequent clinical studies [22, 23]. In our previous preliminary study, we observed that QCHS inhibited the activation of DCs and downregulated the expression of IL-17 in UC [24, 25], but the influence of QCHS on T cells and DCs still needs to be elucidated. Therefore, the aim of the present study was to further investigate whether the DC-mediated Th17/Treg balance is the mechanism by which QCHS ameliorates UC.

## Methods

### Animals and reagents

Sippr-BK Laboratory Animal Co., Ltd. (Shanghai, China) provided male C57BL/6 mice (20 ± 3 g). The mice were kept in standard cages with specific pathogen-free conditions. The animal experiments were subject to approval by the Committee for Animal Research of Nanjing University of Chinese Medicine. Mice were euthanized by an overdose of 5% isoflurane. Isoflurane exposure was continued after 1 min of breathing stoppage followed by cervical dislocation for confirmation of euthanasia. QCHS was provided by the Affiliated Hospital of Nanjing University of Chinese Medicine. The extraction methodology of QCHS has previously been reported [24, 26]. Sulfasalazine (SASP) was purchased from Sine Pharmaceutical Co., Ltd. (Shanghai, China).

### Generation of DCs

Bone marrow-derived DCs were generated from bone marrow as describing [27]. First isolated bone marrow in mice, then lysed red blood cells. 1 × 10^6^/ml bone marrow cells were co-cultured in RPMI-1640 medium with 20 ng/ml GM-CSF, 1% PS, 10%FBS, and 10 ng/ml IL-4 to get immature DCs (imDCs). After 48 h, Bone marrow-derived DCs were grouped to imDCs control group, LPS-treated imDCs group, QCHS-treated imDCs group, and QCHS+LPS-treated imDCs group. LPS (1 μg/ml) and QCHS (10 μg/ml) were used to treat imDCs separately or simultaneously. The supernatants were collected after 36 h.

### Induction of experimental colitis in mice

TNBS was dissolved with saline and alcohol. To establish the experimental colitis mice model, 100 mg/kg TNBS was delivered intrarectally by a 3.5F catheter. Forty-two mice were randomly allocated into seven groups and each group had 6 mice: normal control group, TNBS model group, QCHS (3, 6 and 12 g/kg) groups, SASP group and QCHS-treated DC group. The TNBS model and control groups were administered 0.9% saline (10 ml/kg). The QCHS groups received treatment with QCHS (3, 6 and 12 g/kg). And SASP group received 100 mg/kg SASP per day. The drugs were given by gastric gavage once daily. The concentration of 1 × 10^6^ cells/ml QCHS-treated DCs 1 ml injected into mice in QCHS-treated DC group. All groups were administered for 7 days. Body weight, survival rate, and disease activity index (DAI) including percentage of weight loss, loose stool, and fecal occult blood/bloody stool were recorded to analyze the severity of the TNBS-induced colitis. DAI was described as previously [28].

### Histological analysis

The obtained colonic specimens were fixed in 4% formalin, dehydrated with graded ethanol, embedded in paraffin, and cut into 4-μm-thick sections. Then, the sections were stained with hematoxylin and eosin (H&E). The degree of histological damage was scored by two pathologists with blinded manner. The scores were graded according to an established standard [29]: a range from 0 to 3 indicated the amount of inflammation (acute and chronic) and depth of inflammation, and a range from 0 to 4 indicated the amount of crypt damage or regeneration. These changes were also quantified as the percentage involvement of the disease process: (1) 1–25%; (2) 26–50%; (3) 51–75%; (4) 76–100%.

### Immunohistochemistry

Xylene was used to dewax paraffin embedded colonic tissue. The tissue was rehydrated in gradient concentration of ethanol (100–70%) subsequently. Then endogenous peroxidases were removed in 3% hydrogen peroxidase for 15 min at room temperature followed by 1% goat serum albumin for 10 min at room temperature for blocking. The anti-CD103 (Abcam, Cambridge, UK) was used as primary antibody, incubated overnight at 4 °C. Then sections incubated with horseradish peroxidase-conjugated secondary antibody for 60 min at 37 °C. The sections were counterstained with hematoxylin, dehydrated with ascending concentrations of ethanol, cleared in xylene and mounted.

### Flow cytometry analysis (FACS)

Mesenteric lymph nodes (MLNs) were dissected to get mononuclear cells, which were stimulated by 25 ng/ml PMA and 1 μg/ml ionomycin and then incubated with anti-CD4 (FITC) and anti-CD25 (APC) primary antibodies, washed and fixed. Subsequently, anti-Foxp3 (PE) or anti-IL-17A (PE) antibodies were used to stain the cells respectively.

Tregs or Th17 cells were analyzed by flow cytometry using a FACSCalibur cytometer (Becton, Dickinson and Company, CA, USA), according to the manufacturer’s instructions, and analyzed with CellQuest software. Bone marrow-derived DCs cultured with anti-CD45 (PE), anti-CD86 (APC) and anti-MHC-II (FITC) antibodies. FACS was used to phenotype DCs by MHC-II and CD86 expression.

### Quantitative real-time polymerase chain reaction (qRT-PCR)

Total RNA was isolated using TRIzol reagent and reverse transcribed into cDNA using the PrimeScript RT reagent kit with gDNA eraser (TaKaRa Biotechnology, Dalian, China) according to the manufacturer’s instructions. The data were recorded with a StepOnePlus real-time PCR instrument (Applied Biosystems, Foster City, CA, USA). Using β-actin as a reference gene, the 2^-△△Ct^ method was used to calculate relative gene expression levels. Primers were synthesized by Sangon Biotechnology Ltd. (Shanghai, China). Primer sequences are presented in Table 1.

**Table 1:** Primer sequences for qRT-PCR

Primer	5′-3’
IL-12-F	GCTCGCAGCAAAGCAAGGTAA
IL-12-R	CCATGAGTGGAGACACCAGCA
IL-10-F	AGGATGCACATCAAAAGGCTT
IL-10-R	GGCCTCGGTTAGGAAGGATAC
IL-17-F	AGCACACCCGTCTTCTCTC
IL-17-R	GCTGGAGTTCGCACTGTCC
Foxp3-F	CCCATCCCCAGGAGTCTTG
Foxp3-R	ACCATGACTAGGGGCACTGTA
β-actin-F	CATCCGTAAAGACCTCTATGCCAAC
β-actin-R	ATGGAGCCACCGATCCACA

### Enzyme-linked immunosorbent assay (ELISA)

The levels of IL-17, IL-10 and IL-12 in colonic tissue, MLNs and cell suspensions were detected with ELISA kits (Abcam, Cambridge, UK). Samples were homogenized in 1 ml of ice-cold RIPA lysis buffer containing 1% phosphatase inhibitor cocktail and 1% protease inhibitor cocktail. The lysate was centrifuged (15,000 g, 4 °C). After 10 min, the supernatant was transferred to 96-well ELISA plates.

### Western blot analysis

Proteins were separated by 10% SDS-PAGE gels and then transferred to polyvinylidene fluoride membranes. The blots were incubated with the appropriate concentrations of specific antibodies overnight at 4 °C. Then, the membranes were blocked with 5% nonfat milk in Tris-buffered saline for 2 h at room temperature. After three washes, the membranes were probed with an HRP-conjugated secondary antibody at a 1:5000 dilution for 2 h. The gray value of each band was measured and quantified using ECL detection system (Santa Cruz Biotechnology, CA, USA). The β-actin antibody was utilized under the same conditions as the control.

### Statistical analysis

The data are expressed as the mean ± standard deviation (SD). Data in multiple groups were compared and analyzed by one-way ANOVA attended by Tukey’s multiple comparisons test. All statistical analysis was performed with GraphPad Prism 6.0 (GraphPad Software Inc., CA, USA). A value of *P* < 0.05 was considered statistically significant.

## Results

### QCHS ameliorated the inflammatory infiltration in TNBS-induced colitis mice

**Fig. 1:**
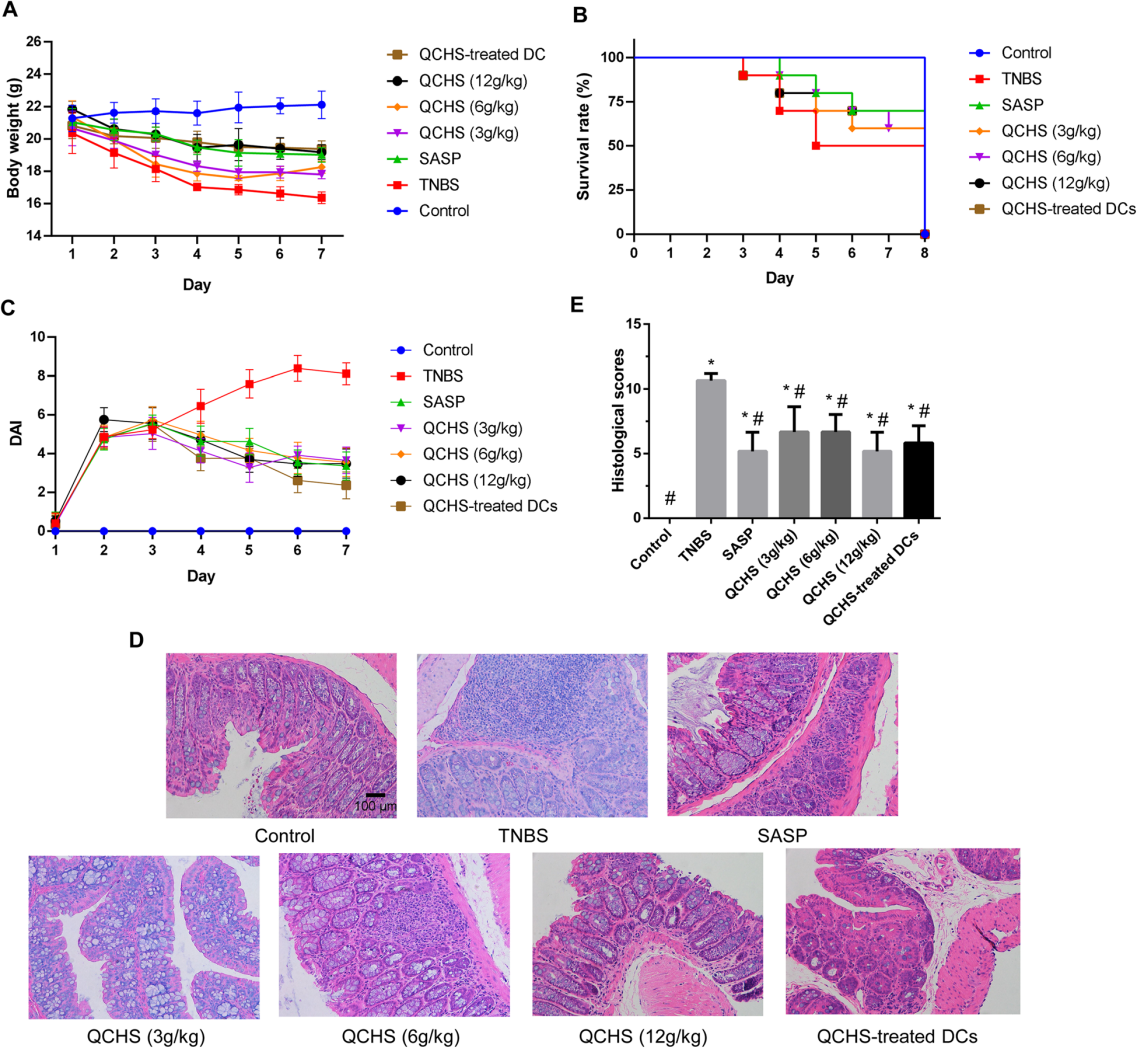
QCHS antagonized TNBS-induced colitis. **a** TNBS-induced experimental colitis caused the loss of body weight in mice. **b** Effects on survival rate in TNBS-induced colitis mice. **c** DAI was recorded daily. **d** Histological changes in colon tissue by H&E staining, detected with a microscope (magnification, 200×). SASP and QCHS (3, 6 and 12 g/kg), as well as QCHS-treated DCs, ameliorated inflammatory infiltration in the colons of colitis mice. **e** Histopathological scores showing that both QCHS and QCHS-treated DCs improved the histology of the colon. The data are expressed as mean ± SD, *n* = 6 per group. *P < 0.05 indicates a significant difference vs the control group. ^#^*P <* 0.05 indicates a significant difference vs the TNBS group

To determine whether QCHS affected inflammation in TNBS-induced colitis, we recorded body weight, survival rate, and DAI. After treatment for 7 days, the body weight of TNBS-induced colitis mice was significantly lower than that of control mice; mice in both the QCHS-treated groups (3, 6 and 12 g/kg) and the SASP group were dramatically heavier than mice in the experimental colitis model group (Fig. 1a). Moreover, after treatment for 7 days, the survival rate of QCHS and SASP groups was higher than that of the experimental colitis model mice group (Fig. 1b). The experimental colitis model mice group exhibited distinctly severe DAI than that in the other groups. Both the QCHS (3, 6 and 12 g/kg) and SASP groups had distinctly reduced DAI than the experimental colitis model mice group (Fig. 1c).

Colonic tissue samples in TNBS model group mice showed conspicuous higher hyperemia and inflammation compared with mice in normal control group. The histology of the colons of TNBS-induced mice was characterized with infiltration by inflammatory cells, loss of crypts, reduced of goblet cells and extensive destruction of the mucosal layer (Fig. 1d). Histological scores in the experimental colitis group increased significantly compared with those in the control group, indicating that the colonic mucosa was severely impaired in the TNBS-induced mice group. To investigate the effects of QCHS on experimental colitis, mice in the QCHS group were treated with QCHS for 7 days before undergoing histological scoring. The QCHS group exhibited remarkable restoration of goblet cells and crypt architecture, reduction in the infiltration of inflammatory cells, consistent with the results of the SASP group. Histological scores in the 3 g/kg, 6 g/kg and 12 g/kg QCHS groups and SASP group were significantly reduced compared with those of the TNBS group (Fig. 1e).

### QCHS modulated the Th17/Treg balance in vivo

**Fig. 2:**
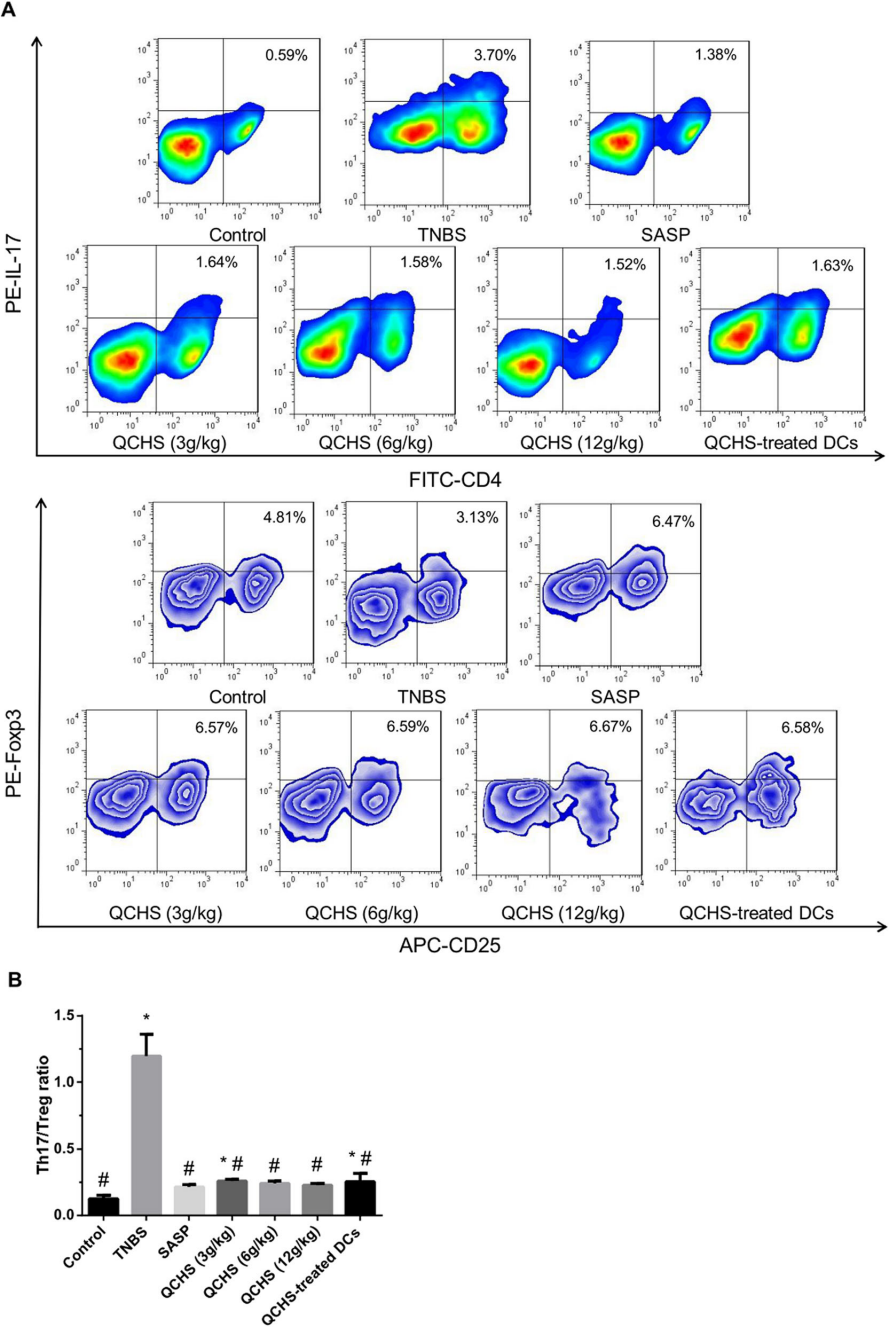
Effects of QCHS on the differentiation of Th17 cells and Tregs in mice with TNBS-induced colitis. After 7 days of administration. **a** The frequencies of Th17 and Tregs in the MLN were analyzed by flow cytometry. **b** QCHS (3, 6 and 12 g/kg) and SASP, as well as QCHS-treated DCs, reduced the ratio of Th17/Treg in TNBS-induced colitis. The data are expressed as mean ± SD, *n =* 6 per group. *P < 0.05 indicates a significant difference vs the control group. ^#^*P* < 0.05 indicates a significant difference vs the TNBS group

To investigate the effect of QCHS on the differentiation of T cells, Tregs and Th17 cells in the MLN were detected by FACS. Figure 2a shows that the percentage of Th17 cells was dramatically increased in TNBS model mice compared with that in the normal control mice. Administration of QCHS (3, 6 and 12 g/kg) reduced the percentage of Th17 cells in the experimental colitis mice, as did treatment with SASP. Moreover, the frequency of Th17 was decreased in a QCHS dose-dependent pattern. The frequency of CD4^+^CD25^+^Foxp3^+^ Tregs was reduced in the experimental colitis model, however the administration of QCHS (3, 6 and 12 g/kg) or SASP markedly upregulated this percentage in the colitis model mice. Both the QCHS (3, 6 and 12 g/kg) and SASP groups exhibited dramatically lower Th17/Treg ratios than those of the TNBS group (Fig. 2b).

**Fig. 3:**
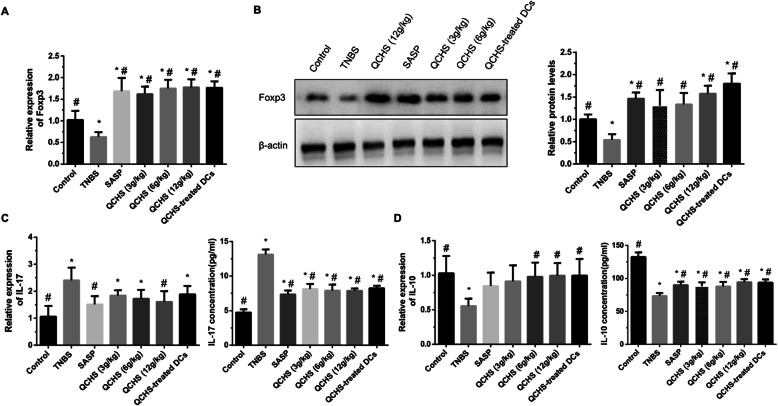
Effects of QCHS on the expression of Treg- and Th17-associated transcription factors and cytokines in colonic tissues from the TNBS-induced experimental colitis mice. **a** and **b** The mRNA and protein levels of Foxp3 were analyzed by qRT-PCR and Western blotting, respectively. **c** and **d** The concentrations of IL-17 and IL-10 were measured by qRT-PCR and ELISA. Both QCHS and QCHS-treated DCs facilitated the production of Foxp3 and IL-10 and restrained the production of IL-17. All values are mean ± SD, *n =* 6 per group. *P < 0.05 indicates a significant difference vs the control group. ^#^*P* < 0.05 indicates a significant difference vs the TNBS group

As shown in Fig. 3a and b, the mRNA and protein expression of Foxp3 in colonic tissue was significantly reduced in the experimental colitis mice compared with the control mice. However, administration of QCHS (3, 6 and 12 g/kg) or SASP dramatically upregulated the expression of Foxp3 in the TNBS-induced colitis model.

IL-17 and IL-10 were detected by qRT-PCR and ELISA. The mRNA expression of IL-17 in colon from the experimental colitis mice group was significantly increased than that of the normal mice group. However, the concentrations of IL-17 mRNA significantly reduced after the administration of 12 g/kg QCHS or SASP compared with those in the experimental colitis mice group. The concentrations of IL-17 in the QCHS (3, 6 and 12 g/kg) and SASP groups were dramatically decreased than those in the experimental colitis model mice group (Fig. 3c). The mRNA expression of IL-10 was significantly reduced in the TNBS group compared with that in the control group. However, IL-10 mRNA levels significantly increased after the administration of 6 g/kg or 12 g/kg QCHS compared with those in the TNBS group. The concentration of IL-10 was lower in the experimental colitis model mice group compared with the normal control mice group, whereas was dramatically upregulated after treatment of QCHS (3, 6 and 12 g/kg) or SASP (Fig. 3d).

### QCHS inhibited DC maturation in vivo and in vitro

DCs expressing CD103 in mice drive T cell activation [30]. DCs also express costimulatory molecule CD86 and MHC-II. Mature DCs have a high concentrations of CD103, CD86 and MHC-II.

**Fig. 4:**
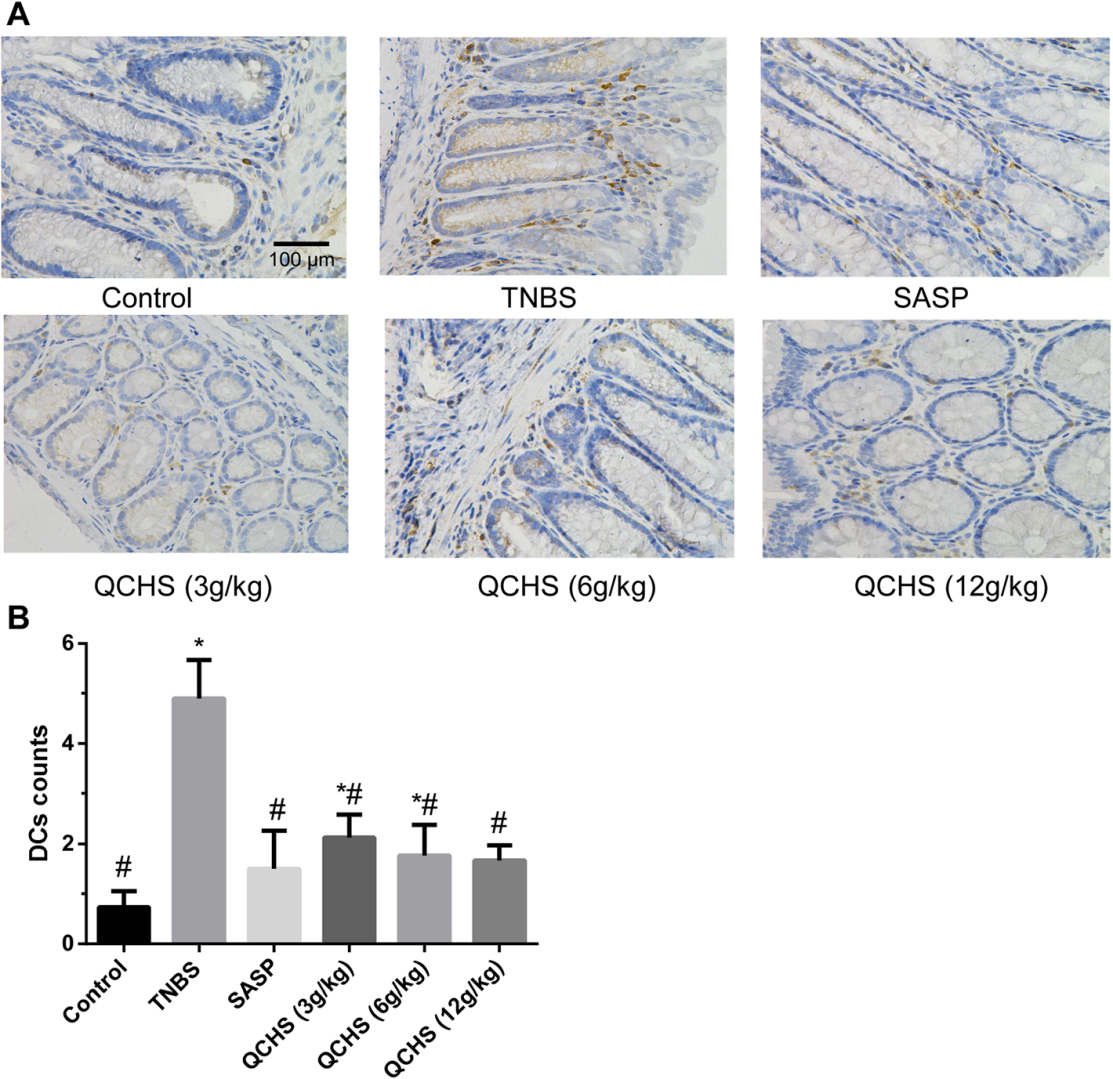
QCHS reduced mature DCs in experimental colitis mice. **a** Staining of DCs was performed with specific marker CD103 (brown) (magnification, 400×). **b** Both QCHS and SASP downregulated CD103^+^ DC counts. The data are expressed as mean ± SD, *n =* 6 per group. *P < 0.05 indicates a significant difference vs the control group. ^#^*P* < 0.05 indicates a significant difference vs the TNBS group

Immunostaining with a CD103 antibody was processed on colon tissue samples. DCs were denser in the tissue of the TNBS model group than the normal control group (Fig. 4a). The DC counts in the QCHS (3, 6 and 12 g/kg) groups and SASP groups were significantly downregulated compared with those in the TNBS group. Only a few CD103^+^ DCs were detected in the 12 g/kg QCHS and SASP groups, which were not significantly different from the control group (Fig. 4b).

**Fig. 5:**
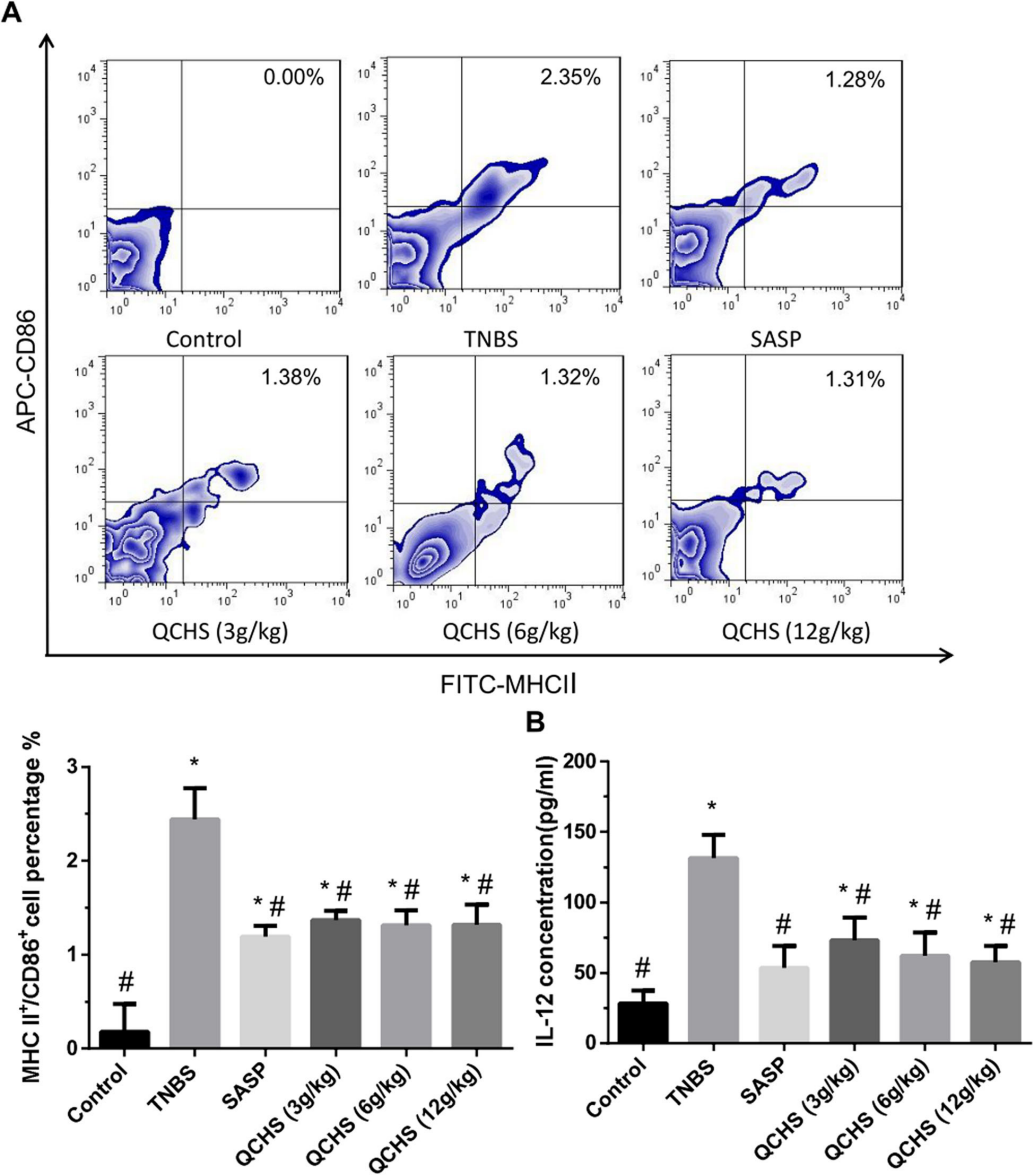
QCHS inhibited the expression of surface molecules and cytokines of DCs in TNBS-induced experimental colitis mice. **a** The frequency of MHC-II^+^CD86^+^ DCs in the MLN was measured by flow cytometry. QCHS (3, 6 and 12 g/kg) and SASP downregulated the percentage of mature DCs. **b** IL-12 was analyzed by ELISA. The concentration of IL-12 was reduced in a QCHS dose-dependent pattern. The data are expressed as mean ± SD, *n =* 6 per group. *P < 0.05 indicates a significant difference vs the control group. ^#^*P* < 0.05 indicates a significant difference vs the TNBS group

To determine DC maturity, the frequency of MHC-II^+^CD86^+^ DCs in the MLN was observed by FACS. The frequency of MHC-II^+^CD86^+^ DCs was markedly increased in the TNBS model group than in the normal control mice group (Fig. 5a). However, administration of QCHS (3, 6 and 12 g/kg) or SASP significantly downregulated the frequency of MHC-II^+^CD86^+^ DCs in the experimental colitis mice.

Mature DCs secrete high concentration of IL-12 [31]. We tested the expression levels of IL-12 in the MLN with ELISA. As shown in Fig. 5b, the concentration of IL-12 was markedly higher in the TNBS model group than in the normal control group but was reduced in response to administration of SASP or QCHS (3, 6 and 12 g/kg) and related to the QCHS dosage.

**Fig. 6:**
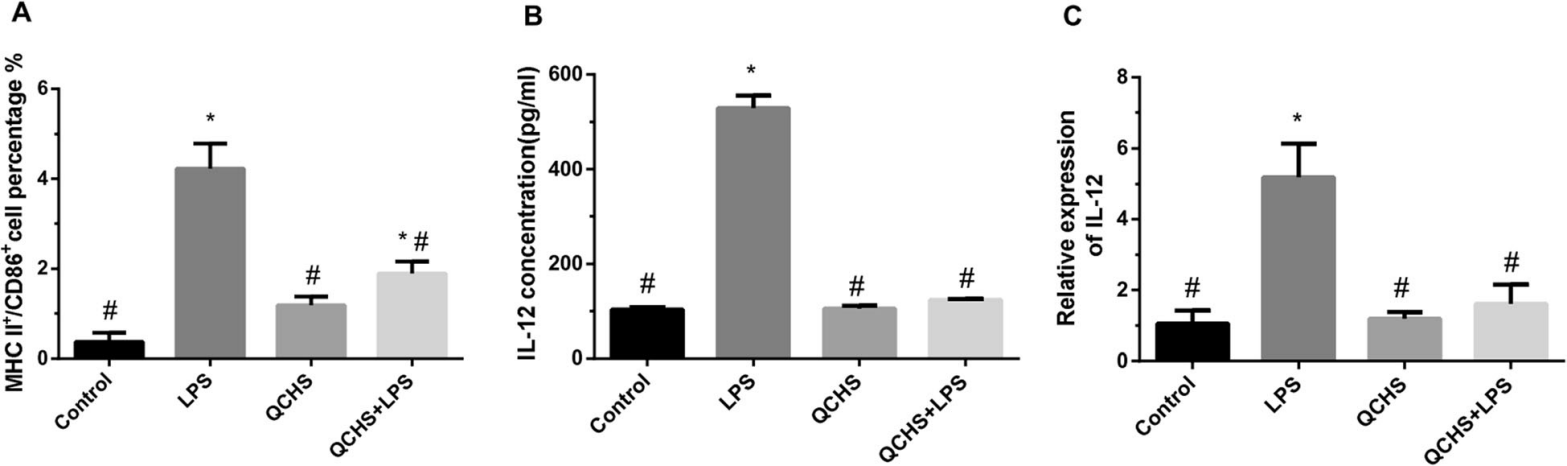
QCHS restrained DC maturation ex vivo. **a** The expression of the surface molecules MHC-II and CD86 in supernatants was assessed by flow cytometry. QCHS decreased the frequency of MHC-II^+^CD86^+^ DCs. **b** and **c** The concentration of IL-12 was analyzed by ELISA and qRT-PCR. DCs treated with QCHS had reduced concentrations and mRNA levels of IL-12. The tests were performed in independent triplicates. All values are the mean ± SD. *P < 0.05 indicates a significant difference vs the control group. ^#^*P* < 0.05 indicates a significant difference vs the LPS group

We next detected the effect of QCHS on DC maturation ex vivo. The frequency of MHC-II^+^CD86^+^ DCs in the LPS group and QCHS+LPS group was markedly increased compared with the imDCs group. The frequency of MHC-II^+^CD86^+^ DCs in the QCHS group and QCHS+LPS group was dramatically decreased than that in the LPS group (Fig. 6a). The expression levels of IL-12 in the supernatants were detected by qRT-PCR and ELISA. As shown in Fig. 6b and c, the LPS group had markedly increased IL-12 mRNA and protein concentrations than the QCHS and QCHS+LPS groups. However, DCs treated with QCHS had significantly lower concentrations and mRNA levels of IL-12 in the QCHS and QCHS+LPS groups compared with results in the LPS groups.

### QCHS-treated DCs ameliorated TNBS-induced colitis by restoring the Th17/Treg balance

QCHS-treated DCs obtained from the in vitro experiment were injected into TNBS-induced colitis model mice. After treatment for 7 days, the QCHS-treated DC group had a higher body weight and significantly lower DAI than the experimental colitis mice group. Colon samples from the QCHS-treated DC group mice exhibited reduced infiltration by inflammatory cells and less disruption of mucosa and epithelial cell layers caused by ulcerations. Histological scores in the QCHS-treated DC group were significantly decreased compared with results in the experimental colitis mice group (Fig. 1).

The frequency of Th17 cells in the MLNs of experimental colitis mice decreased after injection of QCHS-treated DCs, whereas the frequency of CD4^+^CD25^+^Foxp3^+^ Tregs was upregulated. Injection of QCHS-treated DCs in the model mice significantly reduced the Th17/Treg ratio compared with result in the experimental colitis mice (Fig. 2), indicating that QCHS-treated DCs could restore the Th17/Treg balance.

Injection of QCHS-treated DCs significantly upregulated the mRNA and protein expression of Foxp3 in colonic tissue samples compared with that in the TNBS-induced colitis group (Fig. 3a and b). The concentration of IL-17 markedly reduced in the QCHS-treated DC group, whereas the mRNA and protein concentrations of IL-10 markedly increased in the QCHS-treated DC group compared with the experimental colitis model mice group (Fig. 3c and d).

## Discussion

UC is a heterogeneous disease that involves aberrant mucosal immune responses and is characterized by the inappropriate production of a number of proinflammatory cytokines and chemokines [32]. QCHS is formulated according to the TCM pathogenesis of dampness and heat that accumulates in active UC. Previous researches presented the anti-inflammatory properties of QCHS in ameliorating inflammation in the UC model through the MEK/ERK or NF-kappaB/TLR signaling pathway [26, 33]. QCHS also reduces the maturation of DCs and IL-17 levels in UC [24, 25] and upregulates the expression of Foxp3 in experimental colitis mice to promote Treg formation [34]. Paeoniflorin, which is extracted from *Radix Paeoniae Alba* in QCHS, could modulate Tregs in the UC model by DCs [35]. Based on these results, we explored whether QCHS alleviates UC by restoring the DC-mediated Th17/Treg balance.

SASP has been used as a fundamental medicine in UC for decades [36]. We used SASP as a positive control drug in the present study. Our study showed that administration of QCHS or SASP improved histological scores in the experimental colitis mice. QCHS and SASP significantly reduced the Th17/Treg ratio in the experimental colitis mice and downregulated the levels of the Th17 cell-associated cytokine IL-17 and increased the levels of the Treg-associated cytokine IL-10 and transcription factor Foxp3. To explore the reason by which QCHS performs its immunoregulatory properties, we detected the effect of QCHS on DCs in vivo and in vitro. The data indicated that QCHS could reduce CD103^+^ DC numbers in the experimental colitis mice and decrease the frequency of MHC-II^+^CD83^+^ DCs and the levels of IL-12 ex vivo and in vivo, which suggests that QCHS inhibits DC maturation to form immature DCs. Furthermore, to investigate whether QCHS ameliorated UC by regulating the DC-mediated Th17/Treg balance, QCHS-treated DCs were injected into the experimental colitis model. The results showed that, similar to QCHS and SASP, QCHS-treated DCs reduced the histological scores, and the Th17/Treg ratio downregulated the expression of IL-17 and upregulated Foxp3 and IL-10 levels. Our research shows that QCHS alleviates UC by restoring the DC-mediated Th17/Treg balance. Our previously study showed paeoniflorin, the bioactive monoterpene glucoside extracted from the constituents of QCHS, also could modulate the DC-mediated Th17/Treg balance to treat UC [35]. Results of QCHS and paeoniflorin study revealed the immunological mechanism was involved with the treatment of UC. QCHS may exert its immunotherapy effects on UC via restoring immunological tolerance, which concerned with Th17/Treg balance and DC maturation.

Inappropriate T cell responses to intestinal microflora and an increase in epithelial permeability play important roles in the pathological process of UC, which is characterized by the destruction of mucosal immunological tolerance. The principal mechanisms of peripheral immunological tolerance are anergy and the differentiation of naive T cells into Tregs instead of effector T cells [37]. Tregs modulate immune responses by suppressing Th effector cells [38].

The negative regulatory immune function of Tregs is important for maintaining immunological tolerance. Foxp3 is a specific transcription factor in Treg development. The percentage of Foxp3^+^ Tregs, which produce an anti-inflammatory cytokine, IL-10, is decreased in the peripheral blood and intestinal mucosa in patients with IBD [39]. Th17 cells are a newly discovered type of CD4^+^ Th cell that can launch immune responses to break immunological tolerance. By secreting IL-17, Th17 cells mobilize, recruit, and activate neutrophils and macrophages, mediate inflammatory cells in colon tissue, and induce inflammatory responses. Loss of balance between Th17 cells and Tregs is thought to result in the development of UC [40]. Our research revealed that both QCHS and QCHS-treated DCs could upregulate Foxp3 expression and lower the Th17/Treg ratio, which indicates that QCHS and QCHS-treated DCs induced immunological tolerance. QCHS decreased the Th17 differentiation and the expression of IL-12 in this study. The binding of IL-12 family of cytokines to their receptors activates STAT3, which is required for Th17 cell differentiation. The mechanisms by which QCHS decreases the Th17 differentiation may be involved with the IL-12/STAT3 signal pathway, which must be further studied.

DCs play a pivotal role in maintaining immune homeostasis and blocking autoimmune responses, which contribute to the establishment of immunological tolerance. DCs interact with T cells and shape the adaptive immune response. With inflammatory signals from an immune reaction to bacteria or viruses, DCs activate and stimulate T cells to destroy immunological tolerance. However, in the steady state, the antigen-laden DCs polarize the differentiation of naive T cells towards Tregs to keep immunological tolerance. This type of DC that enhances or induces tolerance is a tolerogenic DC [41]. Immunosuppressants inhibit DC maturation to form pharmacologically induced tolerogenic DCs. The mechanisms by which tolerogenic DCs modulate T cell responses are complicated. DCs targeted in vivo with an anti-DEC205 chimeric antibody to deliver myelin oligodendrocyte glycoprotein prevented subsequent experimental autoimmune encephalomyelitis [42]. In a transgenic mouse model of IBD in which hemagglutinin is expressed in the intestinal epithelium, delivery of hemagglutinin to DEC205^+^ DCs prevented IBD induced by hemagglutinin-specific T cells [43]. Vitamin D3 and corticosteroids favor tolerogenic DCs to induce CD4^+^CD25^+^Foxp3^+^ Tregs [44]. Another immunosuppressive drug, rapamycin, which inhibits the protein kinase mTOR, has also been reported to induce CD4^+^CD25^+^Foxp3^+^ Tregs via induction of tolerogenic DCs that stimulate Treg expansion [45]. Genetically engineered tolerogenic DCs can be used to induce Tregs as DCs with genetically enhanced TGF-β, IL-10, or SOCS1 expression [46, 47]. In the present study, QCHS restrained DC maturation, and QCHS-treated DCs promoted the differentiation of naive T cells into Tregs in vivo, the results indicated that the QCHS-treated DCs were a subset of tolerogenic DCs. However, the mechanisms underlying the function of QCHS-treated DCs in inducing Treg differentiation require further clarification.

## Conclusions

Our study detected the influence of QCHS-treated DC on T cells in TNBS-induced colitis mice, and the results suggest that QCHS can alleviate the experimental colitis by restoring the DC-mediated Th17/Treg balance. These results provide a scientific basis for QCHS in the treatment of UC. Our study sheds light on immunotherapy with UC, revealing the immunological mechanisms by which QCHS ameliorates inflammation and identifying novel therapeutic targets. The immunological therapeutic potential of QCHS merits further investigation.

## Data Availability

The datasets used and/or analyzed during the current study are available from the corresponding author on reasonable request.

## Abbreviations

QCHS: Qingchang HuaShi granule
UC: Ulcerative Colitis
IBD: Inflammatory Bowel Disease
TNBS: 2,4,6-Trinitrobenzene Sulfonic Acid
Th17: T helper 17
Tregs: Regulatory T cells
DCs: Dendritic cells
Foxp3: Forkhead box P3
TCM: Traditional Chinese Medicine
SASP: Sulfasalazine
DAI: Disease activity index
MLN: Mesenteric lymph node
